# Computational Analysis of* Gynura bicolor* Bioactive Compounds as Dipeptidyl Peptidase-IV Inhibitor

**DOI:** 10.1155/2017/5124165

**Published:** 2017-08-08

**Authors:** Lina Rozano, Muhammad Redha Abdullah Zawawi, Muhamad Aizuddin Ahmad, Indu Bala Jaganath

**Affiliations:** ^1^Agri-Omics and Bioinformatics Programme, Biotechnology and Nanotechnology Research Centre, Malaysian Agricultural Research and Development Institute, 43400 Selangor, Malaysia; ^2^Institute of Systems Biology (INBIOSIS), Universiti Kebangsaan Malaysia, 43600 Bangi, Malaysia; ^3^Director-General's Office, Malaysian Agricultural Research and Development Institute, 43400 Selangor, Malaysia

## Abstract

The inhibition of dipeptidyl peptidase-IV (DPPIV) is a popular route for the treatment of type-2 diabetes. Commercially available gliptin-based drugs such as sitagliptin, anagliptin, linagliptin, saxagliptin, and alogliptin were specifically developed as DPPIV inhibitors for diabetic patients. The use of* Gynura bicolor* in treating diabetes had been reported in various in vitro experiments. However, an understanding of the inhibitory actions of* G. bicolor* bioactive compounds on DPPIV is still lacking and this may provide crucial information for the development of more potent and natural sources of DPPIV inhibitors. Evaluation of* G. bicolor* bioactive compounds for potent DPPIV inhibitors was computationally conducted using Lead IT and iGEMDOCK software, and the best free-binding energy scores for* G. bicolor* bioactive compounds were evaluated in comparison with the commercial DPPIV inhibitors, sitagliptin, anagliptin, linagliptin, saxagliptin, and alogliptin. Drug-likeness and absorption, distribution, metabolism, and excretion (ADME) analysis were also performed. Based on molecular docking analysis, four of the identified bioactive compounds in* G. bicolor*, 3-caffeoylquinic acid, 5-O-caffeoylquinic acid, 3,4-dicaffeoylquinic acid, and* trans*-5-*p*-coumaroylquinic acid, resulted in lower free-binding energy scores when compared with two of the commercially available gliptin inhibitors. The results revealed that bioactive compounds in* G. bicolor* are potential natural inhibitors of DPPIV.

## 1. Introduction

Type-2 diabetes is a chronic metabolic impairment that affects the quality of life. Currently, diabetes is ranked as the eighth leading cause of death with 1.5 million deaths, and 90% of these are from type-2 diabetes [1]. The main cause of type-2 diabetes is excessive blood glucose and the inability of the body to produce enough insulin, also known as insulin resistance in insulin-targeting tissues such as liver, skeletal muscle, and adipocytes. The body's resistance to insulin causes glucose to remain in the blood, further damaging other organs owing to the high level of sugar, which leads to loss of vision, kidney failure, and cardiovascular diseases.

One way of controlling blood glucose levels is through the inhibition of dipeptidyl peptidase-IV (DPPIV), a serine peptidase responsible for transforming incretins into their inactive metabolites. Incretins or glucagon-like peptide-1 (GLP1) have a role in stimulating glucose-dependent insulin secretion and regulate glycaemia but are short-lived because of DPPIV catalytic activity. Because of this, inhibition of dipeptidyl peptidase-IV increases the level of circulating GLP-1, which then stimulates insulin biosynthesis and secretion, which can reverse the hyperglycemic condition in type-2 diabetes.

The introduction of gliptin-based drugs in 2006 for the treatment of type-2 diabetes has changed the pattern of diabetes medication usage among type-2 diabetes patients [2, 3]. Gliptin drugs increase the concentration of incretin hormones, increasing insulin level in a glucose-dependent manner and decreasing glucagon levels in the circulation. Most diabetic patients opt for gliptin-based pills because they have similar efficacy as sulfonylurea drugs such as metformin. Up until now, eight synthetically developed compounds in the gliptin class have been approved for the treatment of diabetes: sitagliptin, anagliptin, linagliptin, saxagliptin, alogliptin, vildagliptin, teneligliptin, gemigliptin, and dutogliptin [4]. However, wide application among type-2 diabetes patients has led to fatal side effects that relate to high risk of cardiovascular diseases, inflammation of pancreas, allergic reactions, and rheumatoid arthritis [5–9].

In parallel with the discovery and development of chemically synthesized DPPIV inhibitors such as tricyclic heterocycles and fungal synthetic (+)-antroquinonol, the exploitation of plant bioactive compounds for DPPIV inhibitory properties is also underway [10–13]. Novel synthetic compounds have been derived from plant backbone structures, such as compound 55P0110 from quinozolidine alkaloids of the lupine producing plants* Lupinus termis* or* Medicago sativa* [14]. To date, there are more than 20 types of plant compounds reported to have DPPIV inhibitory properties and that have undergone in vitro validations. This includes compounds such as resveratrol, luteolin, apigenin, flavone, and cyanidin 3,5-diglucoside, which can be found in citrus, grapes, soybeans, and aronia berries [15–17]. Other plants species that have DPPIV inhibitory properties that have been demonstrated through in vitro studies are* Urena lobata*,* Fagonia cretica *L.,* Hedera nepalensis *K. Koch,* Senna nigricans*,* Commiphora mukul*,* Emblica officinalis*,* Terminalia arjuna*, and* Smilax china* [18–22].

Traditionally,* Gynura* species have been widely studied for their antidiabetic properties, specifically,* Gynura procumbens* [23–25]. Besides lowering blood glucose levels, it does possess other beneficial physiochemical properties such as anti-inflammatory, antihypertensive, antiulcerogenic, and chemopreventative actions [26–32]. However, studies on* G. bicolor* are not as extensive as* G. procumbens*, but it is reported to have high antihyperglycemic properties because of the presence of flavonoid compounds such as dicaffeoylquinic acid and caffeic acid groups [33, 34].* G. bicolor* also has anti-inflammatory protection, and chemoprevention properties [35–37]. Because of the mass availability of DPPIV inhibitory compounds in plants, dependency on in silico screening for DPPIV inhibitor becomes a crucial part of the discovery of potential DPPIV inhibitors before proceeding to the next stage in the development of drug lead compounds [38, 39].

The aim of this study was to evaluate bioactive compounds in* G. bicolor* as potentially potent inhibitors of DPPIV through molecular docking analysis. The candidate agents discovered can then be further developed as robust DPPIV inhibitors.

## 2. Materials and Method

### 2.1. Plant Extracts and Identification of Bioactive Compounds

*G. bicolor* leaves were collected from the Biotechnology and Nanotechnology Research Centre, Malaysian Agricultural Research and Development Institute (MARDI), Selangor, Malaysia. Plant identification was conducted by Mohd Norfaizal Ghazalli (MARDI) and a voucher specimen of* G. bicolor* (MDI 12809) was deposited in MDI Herbarium, MyGenebank™ Complex, Malaysian Agricultural Research and Development Institute, Selangor, Malaysia. The extraction was performed on ground and freeze-dried samples using methanol extraction. In the methanol extraction, 20 mL of methanol was added to the freeze-dried sample (0.5 g) and the mixture was homogenized for 1 minute followed by vortexing for 30 minutes. The mixture was then centrifuged at 8,900 rpm for 5 minutes at 4°C. The supernatant was filtered with Whatman, number 40 filter paper to remove solid particles from the sample, and 10 mL methanol was added without homogenization. The extracted sample (2.0 mL) was transferred into microcentrifuge tube and dried by vacuum concentration for LCMS-MS/HPLC analysis. Samples were introduced to HPLC for chemical profiling at wavelength 280 nm and 360 nm. This was followed by quantitative identification of compounds in* G. bicolor* using LCMS-MS.

### 2.2. Ligand Preparation

**Figure 1:**
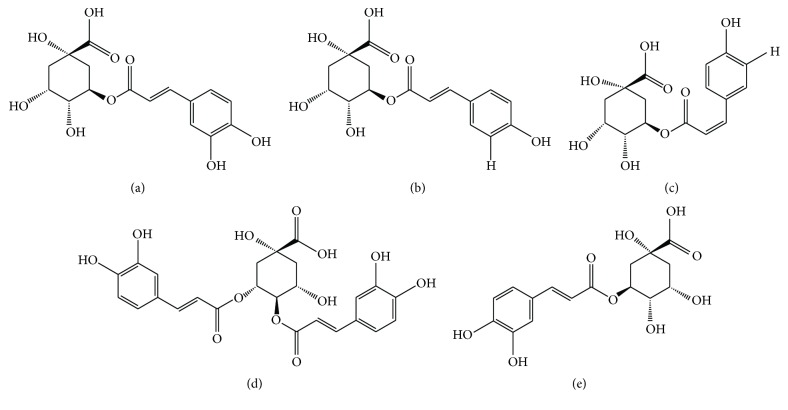
Chemical structures of isolated bioactive compounds from* G. bicolor*. (a) 5-O-caffeoylquinic acid, (b)* trans*-5-*p*-coumaroylquinic acid, (c)* cis*-5-*p*-coumaroylquinic acid, (d) 3,4-dicaffeoylquinic acid, and (e) 3-caffeoylquinic acid.

2D and 3D structure of* G. bicolor* bioactive compounds: 5-O-caffeoylquinic acid,* trans*-5-*p*-coumaroylquinic acid,* cis*-5-*p*-coumaroylquinic acid, 3,4-dicaffeoylquinic acid, and 3-caffeoylquinic acid were generated using ChemDraw (PerkinElmer Inc, Massachusetts, USA) based on LCMS-MS data (Figure 1). All ligand structures file conversions were performed using BIOVIA Discovery Studio Visualizer (Accelrys Software Inc. San Diego, CA) followed by geometrical cleansing. Comparative analysis of the ligands was conducted against five selected gliptin drugs obtained from PubChem database (https://pubchem.ncbi.nlm.nih.gov) [40]: Sitagliptin (PubChem CID: 4369359), Linagliptin (PubChem CID: 10096344), Anagliptin (PubChem CID: 44513473), Saxagliptin (PubChem CID: 11243969), and alogliptin (PubChem CID: 11450633) based on structures obtained from PubChem. Diprotin-A (PubChem CID: 94701), inhibitor of DPPIV receptor, was included in the analysis.

### 2.3. Receptor Preparation

Six 3D protein structure files of dipeptidyl peptidase-IV (DPPIV) with PDB ID: 3WQH, 3W2T, 4A5S, 4FFW, 4PNZ, and 4PV7 were obtained from RCSB Protein Data Bank (http://www.rcsb.org) [41]. The overall stereochemical properties of each DPPIV were assessed based on the information obtained from PDB X-ray Structure Validation Report for each PDB structure, which includes the crystal structure resolution, Wilson *B*-factor, *R*-value, stereochemical parameters, overall percentile scores, and the MolProbity Ramachandran analysis (http://molprobity.biochem.duke.edu/) [42]. The resolution and *R*-values showed the goodness of the protein model being used. The X-ray crystal structure with resolution values of 2.0 Å or less and *R*-values of 0.2 or less are considered acceptable. Structural similarity measurement of the six DPPIV protein structures were conducted using mulPBA web server (http://www.dsimb.inserm.fr/dsimb_tools/mulpba/index.php) based on similarity involving the local backbone [43] and Partial Order Structure Alignment (POSA) web server (http://posa.sanfordburnham.org/) to study structural divergence of the protein structures [44]. The crystal structure of human DPP-IV in complex with a novel heterocyclic DPPIV inhibitor with PDB ID: 4A5S was selected for molecular docking analysis [45]. The active site of DPPIV (PDB ID: 4A5S) was predicted using CASTp Server (http://sts.bioe.uic.edu/castp/) [46], where it scans the protein surfaces for pockets and also interior of proteins for voids followed by further protein functional surfaces identification and spatial pattern characterization using SplitPocket web server (http://pocket.med.wayne.edu/patch/) [47]. Prediction of glycosylation groups for DPPIV (PDB ID: 4A5S) was performed using NetNGlyc 1.0 Server (http://www.cbs.dtu.dk/services/NetNGlyc/) and YinOYang 1.2 Server (http://www.cbs.dtu.dk/services/YinOYang/) [48].

### 2.4. Molecular Docking Simulation

Docking studies were performed using Lead IT software of BioSolve Gmbh FlexX package (http://www.biosolveit.de/FlexX/) [49] and iGEMDOCK software (version 2.1) (http://gemdock.life.nctu.edu.tw/dock/igemdock.php) [50]. Under the FlexX package, the energy minimized DPPIV (PDB ID: 4A5S) receptor and ligands underwent flexible molecular docking analysis. FlexX predicts the geometry of the complex as well as an estimate for the strength of binding by fragmenting the ligand at rotatable bonds and reassembling it within a binding pocket. It contains an optimizer that allows off-grid torsional positions of ligand placement during docking process. Within the torsional degrees of freedom, the procedure moves the atom away from the MIMUMBA grid; the cost function to be minimized is the currently valid scoring function. The protein was prepared in Lead IT using default settings. FlexX binding site analysis included all complete residues with at least one atom within a distance of up to 6.5 Å with respect to the reference ligand. Prior to the docking process, both ligands and DPPIV were prepared and were assigned bonds, bond orders, explicit hydrogens, charges, and flexible torsions. Using a buildup algorithm, the ligands were flexibly located into the protein active site. This is being done through the superposing interaction points of the selected base fragment and the protein active site. The clash factor was set to 0.6. Other parameters were kept as default. The base fragment was then incrementally built up to the complete compound by modeling the ligand flexibility with a torsion library for the added components. The correctness of the protein preparation step was checked by a self-docking process in which the cocrystalized ligand was redocked in the receptor. The root mean square deviation (RMSD) was less than 2.0 Å compared to the reference structure. Up to 200 poses were generated for each compound using FlexX package. The next round of docking studies involves the use of iGEMDOCK software, an integrated virtual screening environment which utilizes postscreening analysis with pharmacological interactions through Generic evolutionary algorithm (GA) and an empirical scoring function. In iGEMDOCK, standard flexible docking (normal) option was selected to perform molecular docking analysis with population size of 200, 70 generations, and 2 solutions. A comparative analysis of the plant metabolites was conducted with the molecular docking scores of DPPIV with commercial drugs. The potent inhibitors for DPPIV were selected based on having the least free-binding energy values.

### 2.5. Drug-Likeness and ADME

The metabolites were analyzed for drug-relevant properties based on “Lipinski's rule of five” and bioactivity prediction using the Molinspiration web server [51]. Further ADME prediction was also conducted using the PreADMET web server (http://preadmet.bmdrc.kr) [52], where the risk of toxicity upon consumption of compounds can be predicted. Four ADME properties of* G. bicolor* bioactive compounds were tested: blood-brain barrier (BBB), human intestinal absorption, Caco-2 cell model, and plasma protein binding ability. BBB was represented as BB = [Brain]/[Blood] or log⁡BB in predicting whether compounds pass across the blood-brain barrier [53]. Prediction of HIA used chemical structures at pH 7.4 and shows the sum of bioavailability and absorption evaluated from the ratio of excretion (or cumulative excretion) in urine, bile, and feces based on percentage values (% HIA) [54, 55]. The Caco-2 cell model is derived from human colon adenocarcinoma and possesses multiple drug transport pathways through the intestinal epithelium. We applied it as a reliable in vitro model for the prediction of oral drug absorption (*P*_Caco-2_ (nm/sec)) [56]. PPB predicts the percentage of compounds bound in plasma protein as in vitro data in humans, which influences the action, disposition, and efficacy of compounds (% PPB).

## 3. Results 

### 3.1. Profiling and Identification of* G. bicolor* Bioactive Compounds

**Figure 2:**
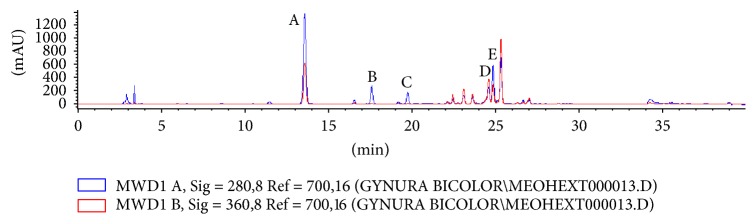
Chromatograms of* G. bicolor* leaf samples using HPLC. Five major peaks were observed based on different elution time with peak A identified as 5-O-caffeoylquinic acid, B* trans*-5-*p*-coumaroylquinic acid, C* cis*-5-*p*-coumaroylquinic acid, D 3,4-dicaffeoylquinic acid, and E 3-caffeoylquinic acid.

**Figure 3:**
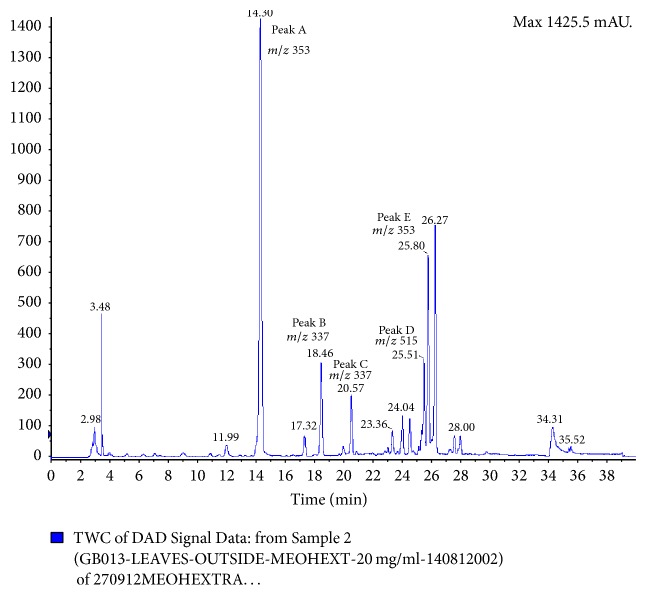
Chromatograms of* G. bicolor* leaf samples with* m/z* ratio using LCMS-MS. Five major peaks were observed based on detection at 280 nm wavelength with peak A identified as 5-O-caffeoylquinic acid, B* trans*-5-*p*-coumaroylquinic acid, C* cis*-5-*p*-coumaroylquinic acid, D 3,4-dicaffeoylquinic acid, and E 3-caffeoylquinic acid.

To profile and identify bioactive compounds in* G. bicolor*, plant extracts were subjected to HPLC and LCMS-MS analysis. HPLC analysis resulted in the observation of five major peaks: A, B, C, D, and E, with peak A being the most abundance followed by peaks E, D, B, and C (Figure 2). Five major peaks were also observed in LCMS-MS chromatogram with detection at 280 nm (Figure 3). Elution time for peaks A, B, C, D, and E was recorded at *t*_*R*_ 14.30, 18.46, 20.47, 25.51, and 25.80 minutes, respectively, as described in Table 1.* G. bicolor* compounds were identified based on* m/z* values of each peak. Peaks A, B, C, D, and E, were identified as 5-O-caffeoylquinic acid,* trans*-5-*p*-coumaroylquinic acid,* cis*-5-*p*-coumaroylquinic acid, 3,4-dicaffeoylquinic acid, and 3-caffeoylquinic acid, respectively (Table 1).

### 3.2. DPPIV Receptor Selection for Docking

**Figure 4:**
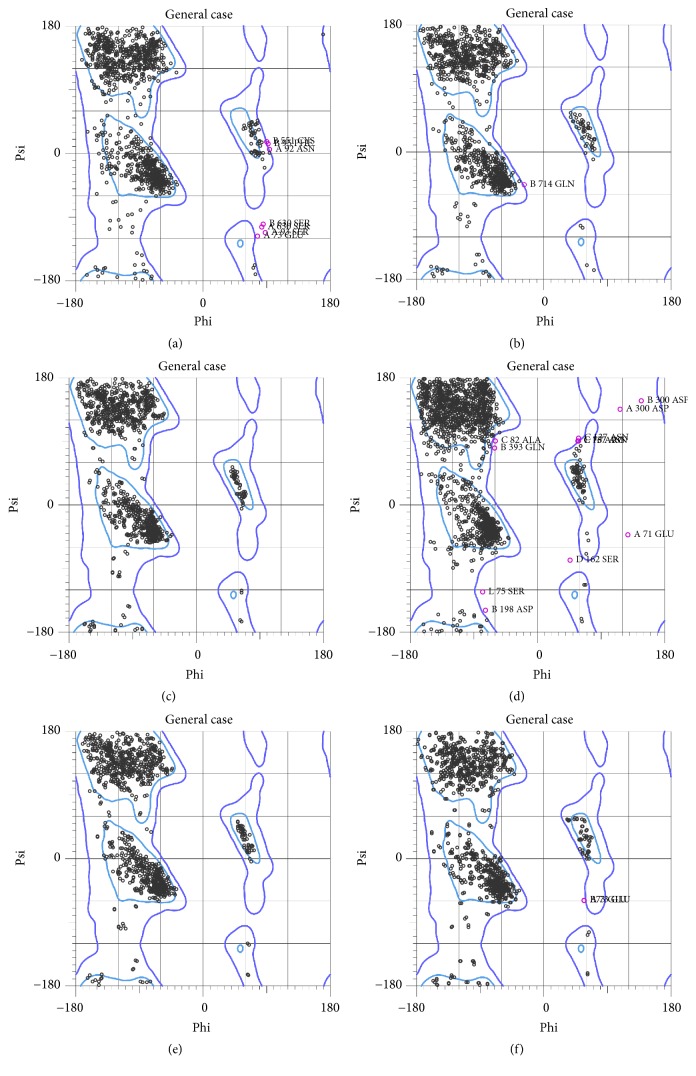
Ramachandran plot analysis for dipeptidyl peptidase-IV receptors obtained from Protein Data Bank based on PDB ID. (a) 3WQH, (b) 3W2T, (c) 4A5S, (d) 4FFW, (e) 4PNZ, and (f) 4PV7.

Determinations of DPPIV receptor for molecular docking process were based on the evaluation of six protein crystal structures obtained from the PDB with PDB ID: 3WQH, 3W2T, 4A5S, 4FFW, 4PNZ, and 4PV7.  Table 2 displays comparison of the six DPPIV receptors according to their crystal structure resolution, Wilson *B*-factor, *R*-value, and Ramanchandran plot values. This also includes information on Ramachandran plots for each structure (Figure 4). Analysis results for the six DPPIV receptors crystal structure similarity, which measures the alignment score, *N*_rms_, *N*_gdt_, RMSD of core, and *N*_3.5_, were displayed in Supplementary Figures  1 and 2, in Supplementary Material, available online at https://doi.org/10.1155/2017/5124165. The DPPIV structure with PDB ID: 4A5S was selected for further molecular docking with* G. bicolor* compounds. Identification of 4A5S ligand binding site of 4A5S was made according to the measurement of the largest identified pocket (pocket 184) with volume area of 19238 Å^3^ and area 6863.7 Å^2^ obtained from CASTp analysis. This was further confirmed with SplitPocket analysis (Supplementary Table  1).

### 3.3. Free-Binding Energy of* G. bicolor* Compounds

To obtain free-binding energy between* G. bicolor* compounds and DPPIV receptor, molecular docking analysis was conducted using Lead IT and iGEMDOCK. Gliptin drugs and diprotin-A, known inhibitors of DPPIV receptor, were also included in the docking analysis for comparison. Molecular docking analysis for all compounds resulted in free-binding energy ranging from as low as −31.8807 KJ/mol to −22.2267 KJ/mol using Lead IT, as presented in Table 3.

As for iGEMDOCK analysis, 3,4-dicaffeoylquinic acid is the top most in the rank with −149.9 kcal/mole followed by 5-O-caffeoylquinic acid,* trans*-5-*p*-coumaroylquinic acid, and 3-caffeoylquinic acid (Table 4). For both docking analysis, highest negative scores indicate a better active compound. All* G. bicolor* compounds and the reference inhibitor, diprotin-A, show electrostatic interaction ranging from −2.81 Kcal/mol to 2.17 Kcal/mol with no electrostatic energy seen in any of the gliptin drugs.

**Figure 5:**
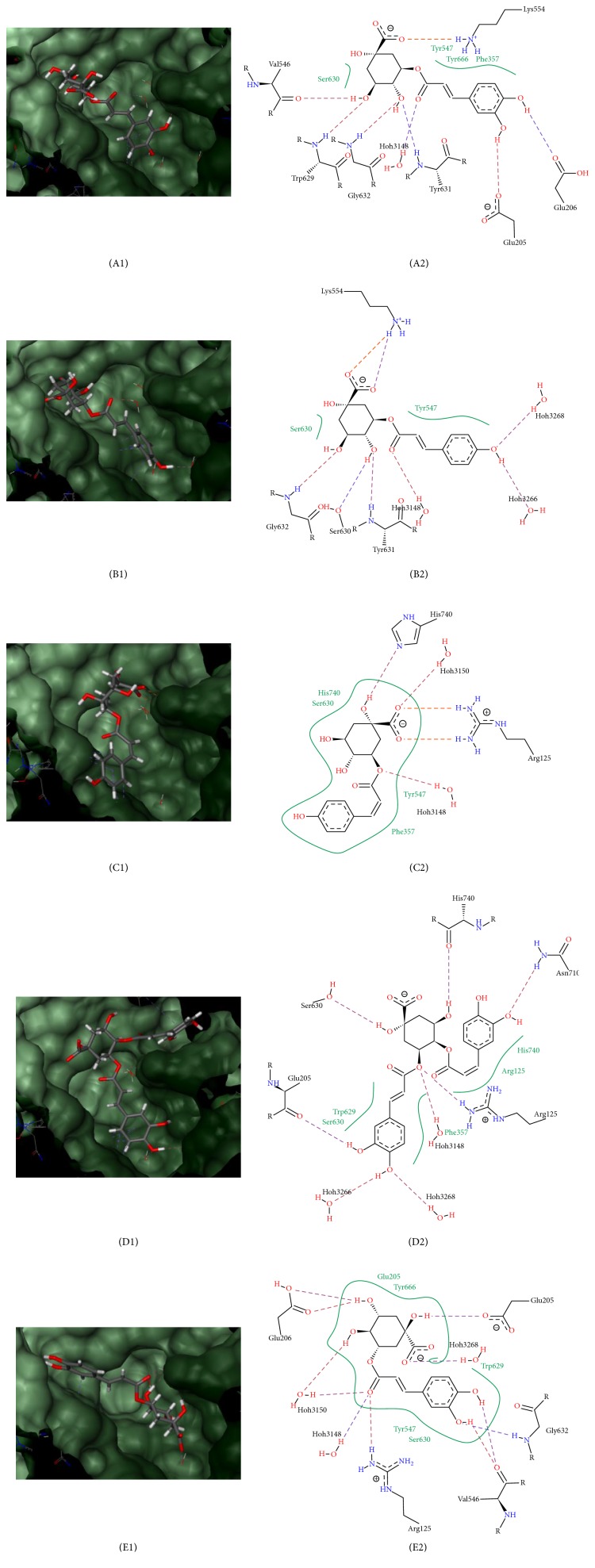
Key interactions of* G. bicolor* compounds with binding sites of DPPIV receptor. (A1/A2) 5-O-caffeoylquinic acid, (B1/B2)* trans*-5-*p*-coumaroylquinic acid, (C1/C2)* cis*-5*-p*-coumaroylquinic acid, (D1/D2) 3,4-dicaffeoylquinic acid, and (E1/E2) 3-caffeoylquinic acid.

**Figure 6:**
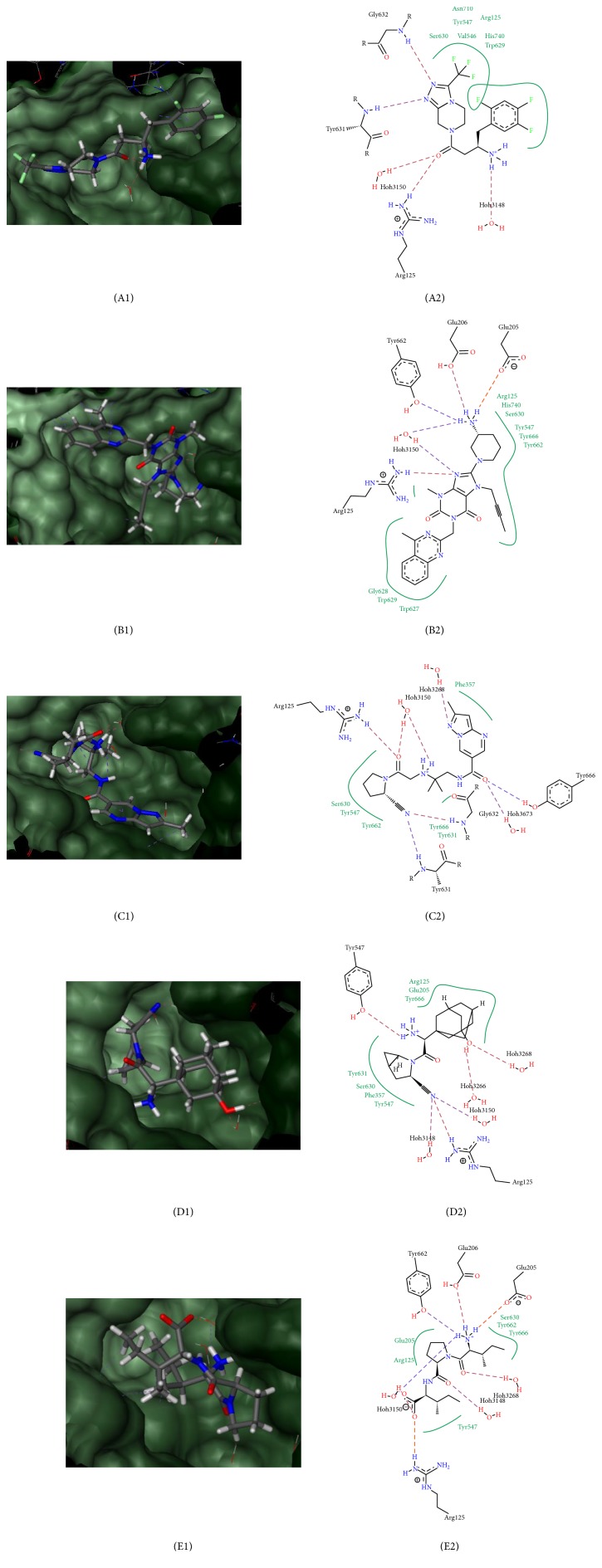
Key interactions of gliptin inhibitor compounds with binding sites of DPPIV receptor. (A1/A2) Sitagliptin, (B1/B2) linagliptin, (C1/C2) anagliptin, (D1/D2) saxagliptin, and (E1/E2) diprotin-A.

To understand the mode of action on DPPIV inhibition through the molecular docking process, binding poses for each structure were studied. Binding pose of* G. bicolor* compounds, together with gliptin drugs and diprotin-A, is indicated in Figures 5 and 6, showing that all compounds interact closely with key residues of sites S1, S2, and S3 within DPPIV receptor pockets.

Results showed that the binding energies of* G. bicolor* compounds with the DPPIV receptor ranged from as low as −29.0750 KJ/mol up to −22.2267 KJ/mol (Table 3). The data were compared with binding energies of gliptin drugs and diprotin-A with the DPPIV receptor. Anagliptin had the lowest binding energy of −31.8807 KJ/mol, while* cis*-5-*p*-coumaroylquinic acid has the highest binding energy of −22.2267 KJ/mol. In our study, molecular docking of* G. bicolor* compounds with 4A5S showed that binding interaction formed with quinic acid of the plant compounds through establishment of hydrogen bond with residues in any of the three DPPIV binding pocket regions, S1, S2, and S3. Plant compound structures were also observed to be located in the hydrophobic regions of DPPIV pockets as observed by the green line (Figure 5).

As observed in Figure 5(A2), 5-O-caffeoylquinic acid of* G. bicolor* forms 8 hydrogen bonds with DPPIV residues. The quinic acid structure resides in S1 pocket of Ser630, while the caffeic acid structure in S3 region interacts with residue Phe357. 3,4-Dicaffeoylquinic acid docking involves interaction of S1 and S3 pockets involving Ser630, His740, and Phe357 residues with its quinic acid structure, while two of its caffeic acid structures interact with the S1 hydrophobic region and Asn710 of S1 pocket. A total of 8 hydrogen bonds was formed. As for* trans*-5-*p*-coumaroylquinic acid, 8 hydrogen bonds were also formed. Its structure differs from the caffeoylquinic acid backbone, with caffeic acid being replaced by coumaric acid. In this case, the coumaric acid did not interact with any of its major pocket regions, but quinic acid resides in S1 hydrophobic region and interacts with Phe357 from S3 pocket region. It was observed that 5-O-caffeoylquinic acid,* trans*-5-*p*-coumaroylquinic acid, and 3-caffeoylquinic acid docked well in the S1 and S3 region of DPPIV receptor unlike 3,4-dicaffeoylquinic acid and* cis*-5-*p*-coumaroylquinic acid. As for the least ranked compound,* cis*-5-*p*-coumaroylquinic acid, only 4 hydrogen bonds were formed between the compounds of DPPIV residues. Its coumaric acid structure resides in the S3 hydrophobic region of Phe357, and quinic acid overlaps in the S1 pockets of both Ser630 and His740 residues.

In comparison with the amino acid interaction of gliptin drugs, anagliptin with the lowest free-binding energy of −31.8807 KJ/mol forms a total of 8 hydrogen bonds with DPPIV residues, and the structure forms interactions with all three active site pockets, S1, S2, and S3, through interaction with residues Phe357, Ser630, and Tyr662 (Figure 6).

### 3.4. Drug-Likeness, ADME, and Bioactivity Prediction

To assess drug-likeness properties of* G. bicolor* compounds, each compound was analyzed using the Molinspiration web server (http://www.molinspiration.com/). Two of the compounds,* trans*-5-*p*-coumaroylquinic acid and* cis*-5-*p*-coumaroylquinic acid, fulfil drug-relevant properties based on “Lipinski's rule of five” as having molecular mass less than 500 Daltons, high lipophilicity (Log⁡*P* less than 5), less than 5 hydrogen bond donors, less than 10 hydrogen bond acceptors, and molar refractivity between 40 and 130. 5-O-caffeoylquinic acid and 3-caffeoylquinic acid have one violation each while 3,4-dicaffeoylquinic acid is with three violations (Table 5).

To predict ADME properties of each* G. bicolor* compound, the compounds were applied to preADMET web server (https://preadmet.bmdrc.kr/). The four ADME results are displayed in Table 6.

## 4. Discussion

This study reported three major findings: (1) the identification of three major compounds, 5-O-caffeoylquinic acid, 3,4-dicaffeoylquinic acid, and* cis*-5-*p*-coumaroylquinic acid, not yet reported before in* G. bicolor*; (2) the prediction of lower free-binding energy scores of four of* G. bicolor* compounds, 3-caffeoylquinic acid, 5-O-caffeoylquinic acid, 3,4-dicaffeoylquinic acid, and* trans*-5-*p*-coumaroylquinic acid when compared to the commercially available gliptin inhibitors; and (3) the computational investigation of DPPIV receptors in relation to protein-ligand binding.

*G. bicolor* is a local herb widely grown in tropical climate countries. Its major compound comprises caffeoylquinic acid backbone, extensively studied for its antidiabetic properties [57, 58]. The identification of 3-caffeoylquinic acid and* trans*-5-*p-*coumaroylquinic acid in* G. bicolor *had been previously reported, while the other three compounds, 5-O-caffeoylquinic acid, 3,4-dicaffeoylquinic acid, and* cis*-5-*p*-coumaroylquinic acid, have not been reported before in* G. bicolor* (Table 1) [33]. 5-O-Caffeoylquinic acid and 3-caffeoylquinic acid, being two of the most abundant compounds discovered in* G. bicolor*, are a well-known chlorogenic acids that have also been observed in coffee and are well studied for their antidiabetic properties [59–61]. However, unlike in coffee, there is no caffeine in* G. bicolor* which makes it free from the side effects that comes with caffeine.

In this study, the molecular docking procedure was aimed at identifying individual poses and the free-binding energy of* G. bicolor* compounds that may bind to the DPPIV active site. Based on the free-binding energy generated from Lead IT, the gliptin drug anagliptin ranked as having the best inhibitory effects towards DPPIV, while* cis*-5-*p*-coumaroylquinic acid ranked as having the least inhibitory effects with free-binding energy of −31.8807 KJ/mol and −22.2267 KJ/mol, respectively. The second-best free-binding energy score was for diprotin-A, followed by alogliptin, 3-caffeoylquinic acid, linagliptin, 3,4-dicaffeoylquinic acid, 5-O-caffeoylquinic acid,* trans*-5-*p*-coumaroylquinic acid, sitagliptin, saxagliptin, and* cis*-5-*p*-coumaroylquinic acid. These data showed that* G. bicolor* bioactive compounds have comparable free-binding energy as observed for gliptin drugs. Gliptin drugs have been widely used as a positive control for DPPIV inhibitory experiments [10, 62]. Among the gliptin drugs, anagliptin is believed to have the best half-maximal inhibitory concentration (IC_50_) values when compared with sitagliptin and alogliptin [63]. This information correlates with the obtained free-binding energy where anagliptin has the lowest binding score among other gliptin classes (Table 3). While diprotin-A is a potent DPPIV inhibitor with a Ile-Pro-Ile sequence commonly used as reference compound [17, 64].

The application of iGEMDOCK highlights the various bond energies, such as hydrogen bond (H-Bond), van der Walls (VDW) interaction, and electrostatic energy that occurs between ligand and receptor. The H-Bond interaction in ligand is related to the interaction of the hydrophilic group or the presence of atom with lone pair electron, while VDW interaction is related to lipophilic groups such as aromatic ring, or methyl group. Majority of the* G. bicolor* compounds and gliptin drugs possesses hydrophilic group in the form of hydroxy indicated by lower H-Bond energy than VDW energy (Table 4). The comparison of binding scores from Lead IT and iGEMDOCK shows similarities in the pattern of the free-binding energy for the top four* G. bicolor* compounds, 3-caffeoylquinic acid, 5-O-caffeoylquinic acid, 3,4-dicaffeoylquinic acid, and* trans*-5-*p*-coumaroylquinic acid, which are lower than* cis*-5-*p*-coumaroylquinic acid (Table 4).

In general, the DPPIV receptor is a cell surface glycoprotein receptor. There are nine major N-glycans and eight O-glycan identified in DPPIV, and these were included during the molecular docking process (Supplementary Figure  3). However, the N-glycans did not induce significant changes in protein structure but, rather, may decrease protein dynamics, thus increasing protein stability [65]. Because of this, the receptor selected for molecular docking should preferably be properly glycosylated to obtain intrinsic dynamic property results. Measurement of structural similarity among the six DPPIV receptors shows that each one exhibits very close relationship based on protein 3D structure alignments. *N*_rms_ values were average, with low RSMD of the core (0.5360 Å) that shows that the structures are very similar (Supplementary Figure  1).

Comparison of the crystal structure of DPPIV does provide information on the flexibility of the side chains possibly involved in ligand stabilization. Nevertheless, it should be understood that the distribution of active site residues for the different DPPIVs differs in where the selected 4A5S has the highest distribution of active sites, as observed in Supplementary Table  2. This is due to structural resolution differences that affect the accuracy of docking prediction, since side chains placements depend on the protein structure resolution. Better resolution means more accurate side chain placement. Side chains play important roles in ligand binding because they can cause steric hindrance, and incorrect placement can form false cavities or pockets [66]. With high resolution structures, the atoms are highly ordered and easy to be observed from the electron density map, while at low resolution, 3 Å or higher, only basic contours of protein chains may be observed and atomic structure must be inferred. Flexibility of the side chains in each DPPIV differs since the distribution of active site during the docking process is affected by overall conformational of the protein and ligand to reach a state of stabilization.

There are three major ligand binding subdomains in the DPPIV structure, identified as S1, S2, and S3 [15]. Each site comprises different amino acid residue positions; subdomain S1 with residues Ser630, Asn710, and His740; subdomain S2 with Lys250, Gly260, and Tyr662; and subdomain S3 with residues Ser209, Phe357, and Arg358. The S1 hydrophobic pocket includes catalytic residues and is the primary determinant of substrate specificity. The selection of the largest binding pocket of DPPIV receptor PDB ID: 4A5S for ligand docking was supported by the physicochemical features of the pocket itself. SplitPocket analysis had identified the presence of a split pocket which is detectable when a functional pocket binds to a ligand, substrate, or other proteins or peptides causing the interaction between heterogeneous atoms to reduce the empty space of the pocket and disrupt the integrity of its surface wall (Supplementary Table  1).

During molecular docking simulation,* G. bicolor* compounds and gliptin drugs occupied the same binding pocket of DPPIV PDB ID: 4A5S formed by residues Val656, Val711, Tyr662, Glu205, Tyr547, Trp629, Tyr631, Ser630, and His 740 (Figures 5 and 6). Ser630 and His 740 interact via hydrogen bonding with the compounds while Val656, Val711, Tyr662, Glu205, Tyr547, Trp629, and Tyr631 formed hydrophobic interactions with the compounds [67]. The main interactions in the active site of DPPIV, which is highly contributed by the hydroxyl coordination between Tyr547 and Ser630 by the water molecule, are highly important for the coordinated interactions in the active site [68]. The location of the binding site appears to be highly conserved across the six DPPIVs being investigated. However, disruption of the binding positions in DPPIV had been reported to impact DPPIV enzyme activity [69]. Reported experimental mutations on active site residue Ser630 resulted in negative activity of DPPIV, while mutating residue His740 greatly reduces inhibitor binding ability to the active sites [70].

The precise position of each* G. bicolor* bioactive compound in DPPIV receptors would reveal the points of interaction with DPPIV residues. The binding score seems to be affected by the number of hydrogen bonds formed between the plant compounds and DPPIV residues. Compared to other compounds found in* G. bicolor*, 3-caffeoylquinic acid has the lowest free-binding energy and is considered to have the most potent DPPIV inhibitory effect. This suggests that the position and the interactions of the hydroxyl groups in caffeic acid, quinic acid, and ester structures with DPPIV binding sites are crucial in determining its bioactivity, as revealed in Figure 5. The main backbone of the 3-caffeoylquinic acid structure resides but does not fully enclose itself in the hydrophobic region and forms 11 hydrogen bonds with DPPIV residues. The quinic acid structure of this molecule resides in the S1 pocket at residue Ser630.

In* G. bicolor*, the major bioactive compounds as indicated in this study have a quinic acid structure of either caffeic or coumaric acid structures attached to either a coumaryl or a caffeoyl group. This structure influences interactions with the DPPIV receptor. The inhibitory mechanisms of compounds from* G. bicolor* exerted on DPPIV receptors were proposed to be involved in competitive binding at the same active site engaged by gliptin drugs which are known to be highly selective and competitive DPPIV inhibitors [71]. If a larger portion of the quinic acid group resides in the hydrophobic region, it could sterically hinder the binding formation involving the active sites within the DPPIV receptor, resulting in higher free-binding energy [15]. This can be observed in the molecular docking interaction between* cis*-5-*p*-coumaroylquinic acid and DPPIV as seen in Figure 5. Here, the whole structure that consists of one quinic acid group and the benzene ring of the coumaryl group is fully enclosed in the hydrophobic regions. However, the free-energy binding for* trans* isomer of 5-*p*-coumaroylquinic acid was reported to be lower compared with its* cis* form from both Lead IT and iGEMDOCK (Tables 3 and 4), as only a small section of the functional groups interacts with the hydrophobic region. This finding correlates with reported bioactive form of* trans* and* cis* chemical compounds where the latter are considered as bioinactive forms [72]. It is believed that* cis*-5-*p*-coumaroylquinic acid is less stable than* trans*-5-*p*-coumaroylquinic acid due to increased steric interaction of the substituents in the* cis* isomer.

In the drug-likeness analysis, all* G. bicolor* compounds passed the minimum cut-off value except for 3,4-dicaffeoylquinic acid, as shown in Table 3. All compounds molecular weight ranged from approximately 330 kDa except for 3,4-dicaffeoylquinic acid with 516 kDa owing to the presence of three phenyl rings. In the ADME analysis (Table 6), all five compounds were CNS -inactive and did not have the ability to cross the blood-brain barrier, which is essential, to avoid any CNS-related effects. HIA analysis revealed that all the studied compounds have the ability to be moderately absorbed from the intestine to the bloodstream and have moderate permeability. Most of the bioactive compounds are weakly bound to plasma protein except for 3,4-dicaffeoylquinic acid. 3,4-Dicaffeoylquinic acid possesses a stronger binding capacity to plasma proteins, making it less preferable as a drug candidate as this characteristic would affect diffusion or transport across cell membranes, limiting pharmacological actions.

## 5. Conclusion

The study demonstrated that 3-caffeoylquinic acid, 5-O-caffeoylquinic acid, 3,4-dicaffeoylquinic acid, and* trans*-5-*p*-coumaroylquinic acid compounds isolated from* G. bicolor* possess DPPIV inhibitory activity. These compounds are able to dock well to two of the DPPIV receptor active sites, S1 and S2. Molecular docking evaluation of* G. bicolor* compounds suggested 3-caffeoylquinic acid as a promising candidate with free-binding energy of −29.0750 KJ/mol, which is better than three of the commercially available gliptin drugs, sitagliptin, saxagliptin, and linagliptin. These data suggested the ability of 3-caffeoylquinic acid to dock well in inhibiting the action of the DPPIV receptor in the treatment of type-2 diabetes. Drug-likeness analysis supports the use of 3-caffeoylquinic as a drug lead compound. ADME properties can be taken as best-hit molecule and can be considered for further studies such as QSAR and molecular dynamics. Despite that, results obtained from this study require further validation of the inhibition action of* G. bicolor* compounds towards the DPPIV receptor using in vitro approach. The computational data supports the efficacy of* G. bicolor* compounds as naturally occurring DPPIV inhibitors and could be considered for development as a potent antidiabetic drug.

## Supplementary Material

Structural alignment, pockets, glycosylation and active sites analysis of 3WQH, 3W2T, 4A5S, 4FFW, 4PNZ and 4PV7 receptors. Supplementary Figure 1: mulPBA alignment of crystal structure similarities for 3WQH, 3W2T, 4A5S, 4FFW, 4PNZ and 4PV7 receptors. Supplementary Figure 2: Analysis result on 3WQH, 3W2T, 4A5S, 4FFW, 4PNZ and 4PV7 receptors crystal structure similarity using POSA web server. Supplementary Table 1: CASTp and SplitPocket analysis for 3WQH, 3W2T, 4A5S, 4FFW, 4PNZ and 4PV7 receptors. Supplementary Figure 3: Prediction of N-glycosylation and O-glycosylation sites for 4A5S. Supplementary Table 2: Active site residues for 3WQH, 3W2T, 4A5S, 4FFW, 4PNZ and 4PV7 receptors.

## Figures and Tables

**Table 1:** Characterization of bioactive compounds in *G. bicolor* leaves.

Peak/compound number	*m/z*	Compound	Fragmented ion	HPLC *t*_*R*_
	[M-H]		*m*/*z*	(min)

A	353	5-Caffeoylquinic acid	193, 191, 179, 161, 135	13.5
B	337	*trans*-5-*p*-Coumaroylquinic acid	191, 173, 163, 145, 119, 93	17.5
C	337	*cis*-5-*p*-Coumaroylquinic acid	191, 173, 163, 145, 119, 93	19.8
D	515	3,4-Dicaffeoylquinic acid	353, 335, 191, 179, 173, 135	24.8
E	353	3-Caffeoylquinic acid	191, 179, 161, 135	25.2

**Table 2:** Criteria for selection of DPPIV receptor for molecular docking.

PDB ID	Resolution (Å)	Wilson plot *B* factor (Å^2^)	*R*-value	Phi psi in most favored regions (%)	Ramachandran outliers (%)
3WQH	2.85	57.7	0.227	92.1	0.5
3W2T	2.36	41.9	0.180	95.9	0.1
4A5S	1.62	20.8	0.164	97.0	0.0
4FFW	2.90	74.4	0.287	91.9	1.0
4PNZ	1.90	24.2	0.168	96.8	0.0
4PV7	3.24	114.9	0.223	92.7	0.2

**Table 3:** Calculated binding energy (KJ/mol) of *G. bicolor* compounds, gliptin drugs, and diprotin-A on DPPIV inhibitor (PDB ID: 4A5S) using Lead IT software.

Ligands	Score	Match	Lipo	Ambig	Clash	Rot
5-O-Caffeoylquinic acid	−27.3288	−33.6824	−7.5070	−7.2838	5.9444	9.800
*trans*-5-*p*-Coumaroylquinic acid	−27.1177	−31.2300	−7.3174	−7.9231	2.7528	11.200
*cis*-5-*p*-Coumaroylquinic acid	−22.2267	−32.6442	−3.4542	−4.6060	3.2777	9.800
3,4-Dicaffeoylquinic acid	−27.1703	−33.4586	−8.8033	−8.8360	3.1276	15.400
3-Caffeoylquinic acid	−29.0750	−33.9425	−6.8680	−7.5393	2.6748	11.200
Sitagliptin	−25.0414	−23.2457	−10.2325	−9.7454	4.3823	8.400
Linagliptin	−27.5774	−26.5346	−10.3421	−9.2890	6.1882	7.000
Anagliptin	−31.8807	−31.9031	−12.7153	−7.2000	7.5376	7.000
Saxagliptin	−23.4595	−23.8361	−6.4052	−8.3097	4.0915	5.600
Alogliptin	−29.7682	−25.9364	−8.3468	−8.2281	3.1431	4.200
Diprotin-A	−30.6185	−36.7587	−7.6816	−8.5830	4.4048	12.600

Score: the total score of the docking solution. Match: contribution of the matched interacting groups. Lipo: contribution of the lipophilic contact area. Ambig: contribution of the lipophilic-hydrophilic or ambiguous contact area. Clash: contribution of the clash penalty. Rot: ligand conformational entropy score (Rot).

**Table 4:** Calculated total energy (Kcal/mol) of *G. bicolor* compounds, gliptin drugs, and diprotin-A on DPPIV inhibitor (PDB ID: 4A5S) using iGEMDOCK software.

Ligands	Total energy	H-bond	VDW	Elec	Rank
5-O-Caffeoylquinic acid	−134.3	−44.81	−86.65	−2.81	2
*trans*-5-*p*-Coumaroylquinic acid	−118.2	−31.7	−83.24	−3.3	3
*cis*-5-*p*-Coumaroylquinic acid	−110.1	−35.87	−76.41	2.17	7
3,4-Dicaffeoylquinic acid	−149.9	−43.64	−102.79	−3.51	1
3-Caffeoylquinic acid	−114.7	−38.33	−74.7	−1.71	4
Sitagliptin	−91.2	−11.45	−79.7	0	11
Linagliptin	−113.6	−22.47	−91.1	0	5
Anagliptin	−102.2	−25.25	−76.96	0	10
Saxagliptin	−105.1	−22.63	−82.5	0	8
Alogliptin	−112.3	−8.84	−103.47	0	6
Diprotin-A	−104.2	−31.52	−70.61	−2.1	9

Total energy in Kcal/mol. Van der Waals interaction (VDW, Kcal/mol), hydrogen bonding (H-Bond, Kcal/mol), electrostatic interactions (Elec, Kcal/mol), average conpair, and rank (based on total energy). Total energy = VDW + H-Bond + Elec.

**Table 5:** Lipinski's rule of 5 analysis for ligands used in the study.

Compounds	Lipinski's rule of five				
	Molecular weight	Hydrogen bond donor	Hydrogen bond acceptor	Log⁡*P*	Molecular polar surface area (PSA)
5-O-Caffeoylquinic acid	354.31	6	9	−0.45	164.74
*trans*-5-*p*-Coumaroylquinic acid	338.31	5	8	0.04	144.52
*cis*-5-*p*-Coumaroylquinic acid	338.31	5	8	0.04	144.52
3,4-Dicaffeoylquinic acid	516.46	7	12	1.21	211.28
3-Caffeoylquinic acid	354.31	6	9	−0.45	164.74

**Table 6:** ADME properties of *G. bicolor* bioactive compounds.

ADME	5-O-Caffeoylquinic acid	*trans*-5-*p*-Coumaroylquinic acid	*cis*-5-*p*-Coumaroylquinic acid	3,4-Dicaffeoylquinic acid	3-Caffeoylquinic acid
BBB	0.033661	0.0315336	0.0315336	0.0360627	0.033661
HIA	20.427827	39.827828	39.827828	23.123325	20.427827
CaCo2	18.7168	19.7961	19.7961	19.5286	18.7168
PPB	41.96179	46.246716	46.246716	87.772544	41.96179

## References

[1] (2014). *Global status report on noncommunicable diseases 2014*.

[2] Drucker D. J., Nauck M. A. (2006). The incretin system: glucagon-like peptide-1 receptor agonists and dipeptidyl peptidase-4 inhibitors in type 2 diabetes. *Lancet*.

[3] Lovshin J. A., Drucker D. J. (2009). Incretin-based therapies for type 2 diabetes mellitus. *Nature Reviews Endocrinology*.

[4] Gupta V., Kalra S. (2011). Choosing a Gliptin. *Indian Journal of Endocrinology and Metabolism*.

[5] Doggrell S. A., Dimmitt S. B. (2016). Sitagliptin and other ‘gliptins’ — Why prescribe them?. *Expert Opinion on Pharmacotherapy*.

[6] Food. (2015). Drug administration, FDA warns that DPP-4 inhibitors for type 2 diabetes may cause severe joint pain. *FDA Drug Safety Communication*.

[7] Bolen S. D., Maruthur N. M. (2016). The safety of incretin based drug treatments for type 2 diabetes. *BMJ (Clinical research ed.)*.

[8] Tseng C.-H. (2016). Sitagliptin and pancreatic cancer risk in patients with type 2 diabetes. *European Journal of Clinical Investigation*.

[9] Li L., Li S., Deng K. (2016). Dipeptidyl peptidase-4 inhibitors and risk of heart failure in type 2 diabetes: Systematic review and meta-analysis of randomised and observational studies. *BMJ (Online)*.

[10] Wu W.-L., Hao J., Domalski M. (2016). Discovery of novel tricyclic heterocycles as potent and selective DPP-4 inhibitors for the treatment of type 2 diabetes. *ACS Medicinal Chemistry Letters*.

[11] Cox J. M., Chu H. D., Kuethe J. T. (2016). The discovery of novel 5,6,5- and 5,5,6-tricyclic pyrrolidines as potent and selective DPP-4 inhibitors. *Bioorganic and Medicinal Chemistry Letters*.

[12] Hsu C. Y., Sulake R. S., Huang P.-K. (2015). Synthetic (+)-antroquinonol exhibits dual actions against insulin resistance by triggering AMP kinase and inhibiting dipeptidyl peptidase IV activities. *British Journal of Pharmacology*.

[13] Schwehm C., Li J., Song H., Hu X., Kellam B., Stocks M. J. (2015). Synthesis of new DPP-4 inhibitors based on a novel tricyclic scaffold. *ACS Medicinal Chemistry Letters*.

[14] Brunmair B., Lehner Z., Stadlbauer K. (2015). 55P0110, A novel synthetic compound developed from a plant derived backbone structure, shows promising anti-hyperglycaemic activity in mice. *PLoS ONE*.

[15] Fan J., Johnson M. H., Lila M. A., Yousef G., De Mejia E. G. (2013). Berry and citrus phenolic compounds inhibit dipeptidyl peptidase IV: Implications in diabetes management. *Evidence-based Complementary and Alternative Medicine*.

[16] Yamane T., Kozuka M., Konda D. (2016). Improvement of blood glucose levels and obesity in mice given aronia juice by inhibition of dipeptidyl peptidase IV and *α*-glucosidase. *Journal of Nutritional Biochemistry*.

[17] Gao Y., Zhang Y., Zhu J. (2015). Recent progress in natural products as DPP-4 inhibitors. *Future Medicinal Chemistry*.

[18] Yusuf S., Suleiman A. M., Lawal S. B., Babangida M. S. (2016). Inhibitory activity of fractions of Senna nigricans toward protein tyrosine phosphatase 1B and dipeptidyl peptidase IV. *Journal of Medicinal Plants Research*.

[19] Borde M. K., Mohanty I. R., Suman R. K., Deshmukh Y. A. (2016). Dipeptidyl peptidase-IV inhibitory activities of medicinal plants: Terminalia arjuna, Commiphora mukul, Gymnema sylvestre, Morinda citrifolia, Emblica officinalis. *Asian Journal of Pharmaceutical and Clinical Research*.

[20] Zhao B. T., Le D. D., Nguyen P. H. (2016). PTP1B, *α*-glucosidase, and DPP-IV inhibitory effects for chromene derivatives from the leaves of Smilax china L.. *Chemico-Biological Interactions*.

[21] Saleem S., Jafri L., Haq I. U. (2014). Plants Fagonia cretica L. and Hedera nepalensis K. Koch contain natural compounds with potent dipeptidyl peptidase-4 (DPP-4) inhibitory activity. *Journal of Ethnopharmacology*.

[22] Purnomo Y., Soeatmadji D. W., Sumitro S. B., Widodo M. A. (2015). Anti-diabetic potential of Urena lobata leaf extract through inhibition of dipeptidyl peptidase IV activity. *Asian Pacific Journal of Tropical Biomedicine*.

[23] Mohd Zaidan M. W. A., Shukor S. A., Machap C. (2015). Expression profiling of diabetes-related genes in streptozotocininduced rat after treatment with herbal mixture extract. *Jurnal Intelek*.

[24] Hassan Z., Yam M. F., Ahmad M., Yusof A. P. M. (2010). Antidiabetic properties and mechanism of action of gynura procumbens water extract in streptozotocin-induced diabetic rats. *Molecules*.

[25] Algariri K., Meng K. Y., Atangwho I. J. (2013). Hypoglycemic and anti-hyperglycemic study of Gynura procumbens leaf extracts. *Asian Pacific Journal of Tropical Biomedicine*.

[26] Kim M.-J., Lee H. J., Wiryowidagdo S., Kim H. K. (2006). Antihypertensive effects of * Gynura procumbens* extract in spontaneously hypertensive rats. *Journal of Medicinal Food*.

[27] Mahmood A. A., Mariod A. A., Al-Bayaty F., Abdel-Wahab S. I. (2010). Anti-ulcerogenic activity of * Gynura procumbens* leaf extract against experimentally-induced gastric lesions in rats. *Journal of Medicinal Plants Research*.

[28] Puangpronpitag D., Chaichanadee S., Naowaratwattana W. (2010). Evaluation of nutritional value and antioxidative properties of the medicinal plant Gynura procumbens extract. *Asian Journal of Plant Sciences*.

[29] Agustina D., Wasito W., Haryana S., Supartinah A. (2015). Anticarcinogenesis effect of Gynura procumbens (Lour) Merr on tongue carcinogenesis in 4NQO-induced rat. *Dental Journal (Majalah Kedokteran Gigi)*.

[30] Tan H.-L., Chan K.-G., Pusparajah P., Lee L.-H., Goh B.-H. (2016). Gynura procumbens: An overview of the biological activities. *Frontiers in Pharmacology*.

[31] Shwter A. N., Abdullah N. A., Alshawsh M. A. (2014). Chemoprevention of colonic aberrant crypt foci by Gynura procumbens in rats. *Journal of Ethnopharmacology*.

[32] Li X.-J., Mu Y.-M., Li T.-T. (2015). Gynura procumbens reverses acute and chronic ethanol-induced liver steatosis through MAPK/SREBP-1c-dependent and -independent pathways. *Journal of Agricultural and Food Chemistry*.

[33] Teoh W. Y., Tan H. P., Ling S. K., Abdul Wahab N., Sim K. S. (2016). Phytochemical investigation of Gynura bicolor leaves and cytotoxicity evaluation of the chemical constituents against HCT 116 cells. *Natural Product Research*.

[34] Zhou X., Zhou M., Liu Y., Ye Q., Gu J., Luo G. (2016). Isolation and identification of antioxidant compounds from gynura bicolor stems and leaves. *International Journal of Food Properties*.

[35] Chao C.-Y., Liu W.-H., Wu J.-J., Yin M.-C. (2015). Phytochemical profile, antioxidative and anti-inflammatory potentials of Gynura bicolor DC. *Journal of the Science of Food and Agriculture*.

[36] Teoh W. Y., Sim K. S., Moses Richardson J. S., Abdul Wahab N., Hoe S. Z. (2013). Antioxidant capacity, cytotoxicity, and acute oral toxicity of Gynura bicolor. *Evidence-based Complementary and Alternative Medicine*.

[37] Wu C.-C., Lii C.-K., Liu K.-L., Chen P.-Y., Hsieh S.-L. (2013). Antiinflammatory activity of Gynura bicolor (Hóng Fèng Cài) ether extract through inhibits nuclear factor kappa B activation. *Journal of Traditional and Complementary Medicine*.

[38] Guasch L., Sala E., Ojeda M. J. (2012). Identification of novel human dipeptidyl peptidase-IV inhibitors of natural origin (Part II): in silico prediction in antidiabetic extracts. *PLoS ONE*.

[39] Irwin J. J., Shoichet B. K. (2016). Docking screens for novel ligands conferring new biology. *Journal of Medicinal Chemistry*.

[40] Kim S., Thiessen P. A., Bolton E. E. (2016). PubChem substance and compound databases. *Nucleic Acids Research*.

[41] Berman H. M., Westbrook J., Feng Z. (2000). The protein data bank. *Nucleic Acids Research*.

[42] Chen V. B., Arendall W. B., Headd J. J. (2010). MolProbity: all-atom structure validation for macromolecular crystallography. *Acta Crystallographica Section D: Biological Crystallography*.

[43] Léonard S., Joseph A. P., Srinivasan N., Gelly J.-C., De Brevern A. G. (2014). MulPBA: An efficient multiple protein structure alignment method based on a structural alphabet. *Journal of Biomolecular Structure and Dynamics*.

[44] Li Z., Natarajan P., Ye Y., Hrabe T., Godzik A. (2014). POSA: A user-driven, interactive multiple protein structure alignment server. *Nucleic Acids Research*.

[45] Sutton J. M., Clark D. E., Dunsdon S. J. (2012). Novel heterocyclic DPP-4 inhibitors for the treatment of type 2 diabetes. *Bioorganic & Medicinal Chemistry Letters*.

[46] Dundas J., Ouyang Z., Tseng J., Binkowski A., Turpaz Y., Liang J. (2006). CASTp: computed atlas of surface topography of proteins with structural and topographical mapping of functionally annotated residues. *Nucleic Acids Research*.

[47] Tseng Y. Y., Dupree C., Chen Z. J., Li W.-H. (2009). SplitPocket: Identification of protein functional surfaces and characterization of their spatial patterns. *Nucleic Acids Research*.

[48] Gupta R., Brunak S. (2002). Prediction of glycosylation across the human proteome and the correlation to protein function. *Pacific Symposium on Biocomputing*.

[49] Gastreich M., Lilienthal M., Briem H., Claussen H. (2006). Ultrafast de novo docking combining pharmacophores and combinatorics. *Journal of Computer-Aided Molecular Design*.

[50] Hsu K. C., Chen Y. F., Lin S. R., Yang J. M. (2011). Igemdock: a graphical environment of enhancing gemdock using pharmacological interactions and post-screening analysis. *BMC Bioinformatics*.

[51] Lipinski C. A. (2004). Lead- and drug-like compounds: the rule-of-five revolution. *Drug Discovery Today: Technologies*.

[52] Lee S. K., Lee I. H., Kim H. J., Chang G. S., Chung J. E., No K. T. (2003). The PreADME Approach: Web-based program for rapid prediction of physico-chemical, drug absorption and drug-like properties. EuroQSAR 2002 Designing drugs and crop protectants: processes, problems and solutions. *Blackwell Publishing*.

[53] Ma X.-L., Chen C., Yang J. (2005). Predictive model of blood-brain barrier penetration of organic compounds. *Acta Pharmacologica Sinica*.

[54] Zhao Y. H., Le J., Abraham M. H. (2001). Evaluation of human intestinal absorption data and subsequent derivation of a quantitative structure–activity relationship (QSAR) with the Abraham descriptors. *Journal of Pharmaceutical Sciences*.

[55] Yee S. (1997). In vitro permeability across Caco-2 cells (colonic) can predict in vivo (small intestinal) absorption in man—fact or myth. *Pharmaceutical Research*.

[56] Yamashita S., Furubayashi T., Kataoka M., Sakane T., Sezaki H., Tokuda H. (2000). Optimized conditions for prediction of intestinal drug permeability using Caco-2 cells. *European Journal of Pharmaceutical Sciences*.

[57] Wu C., Zhang X., Zhang X. (2014). The caffeoylquinic acid-rich Pandanus tectorius fruit extract increases insulin sensitivity and regulates hepatic glucose and lipid metabolism in diabetic db/db mice. *Journal of Nutritional Biochemistry*.

[58] Chen J., Mangelinckx S., Ma L., Wang Z., Li W., De Kimpe N. (2014). Caffeoylquinic acid derivatives isolated from the aerial parts of Gynura divaricata and their yeast *α*-glucosidase and PTP1B inhibitory activity. *Fitoterapia*.

[59] Crozier T. W. M., Stalmach A., Lean M. E. J., Crozier A. (2012). Espresso coffees, caffeine and chlorogenic acid intake: potential health implications. *Food & Function*.

[60] Higdon J. V., Frei B. (2006). Coffee and health: a review of recent human research. *Critical Reviews in Food Science and Nutrition*.

[61] Lafay S., Morand C., Manach C., Besson C., Scalbert A. (2006). Absorption and metabolism of caffeic acid and chlorogenic acid in the small intestine of rats. *British Journal of Nutrition*.

[62] Kushwaha R. N., Srivastava R., Mishra A. (2015). Design, synthesis, biological screening, and molecular docking studies of piperazine-derived constrained inhibitors of DPP-IV for the treatment of type 2 diabetes. *Chemical Biology and Drug Design*.

[63] Nishio S., Abe M., Ito H. (2015). Anagliptin in the treatment of type 2 diabetes: Safety, efficacy, and patient acceptability. *Diabetes, Metabolic Syndrome and Obesity: Targets and Therapy*.

[64] Shin J. W., Jurisic G., Detmar M. (2008). Lymphatic-specific expression of dipeptidyl peptidase IV and its dual role in lymphatic endothelial function. *Experimental Cell Research*.

[65] Lee H. S., Qi Y., Im W. (2015). Effects of N-glycosylation on protein conformation and dynamics: Protein Data Bank analysis and molecular dynamics simulation study. *Scientific Reports*.

[66] Škrbić T., Badasyan A., Hoang T. X., Podgornik R., Giacometti A. (2016). From polymers to proteins: The effect of side chains and broken symmetry on the formation of secondary structures within a Wang-Landau approach. *Soft Matter*.

[67] Amini Z., Fatemi M. H., Gharaghani S. (2016). Hybrid docking-QSAR studies of DPP-IV inhibition activities of a series of aminomethyl-piperidones. *Computational Biology and Chemistry*.

[68] Pantaleão S. Q., Maltarollo V. G., Araujo S. C., Gertrudes J. C., Honorio K. M. (2015). Molecular docking studies and 2D analyses of DPP-4 inhibitors as candidates in the treatment of diabetes. *Molecular BioSystems*.

[69] Chien C.-H., Huang L.-H., Chou C.-Y. (2004). One site mutation disrupts dimer formation in human DPP-IV proteins. *Journal of Biological Chemistry*.

[70] Metzler W. J., Yanchunas J., Weigelt C. (2008). Involvement of DPP-IV catalytic residues in enzyme-saxagliptin complex formation. *Protein Science*.

[71] Duez H., Cariou B., Staels B. (2012). DPP-4 inhibitors in the treatment of type 2 diabetes. *Biochemical Pharmacology*.

[72] Koga C. C., Andrade J. E., Ferruzzi M. G., Lee Y. (2016). Stability of trans-resveratrol encapsulated in a protein matrix produced using spray drying to uv light stress and simulated gastro-intestinal digestion. *Journal of Food Science*.

