# Supplementary material for: Computational Analysis of Gynura bicolor Bioactive Compounds as Dipeptidyl Peptidase-IV Inhibitor
Source: Adv Bioinformatics. 2017 Aug 8;2017:5124165. doi: 10.1155/2017/5124165 (PMC5591938; doi:10.1155/2017/5124165)
Supplement: Supplementary file 1 — Structural alignment, pockets, glycosylation and active sites analysis of 3WQH, 3W2T, 4A5S, 4FFW, 4PNZ and 4PV7 receptors. Supplementary Figure 1: mulPBA alignment of crystal structure similarities for 3WQH, 3W2T, 4A5S, 4FFW, 4PNZ and 4PV7 receptors. Supplementary Figure 2: Analysis result on 3WQH, 3W2T, 4A5S, 4FFW, 4PNZ and 4PV7 receptors crystal structure similarity using POSA web server. Supplementary Table 1: CASTp and SplitPocket analysis for 3WQH, 3W2T, 4A5S, 4FFW, 4PNZ and 4PV7 receptors. Supplementary Figure 3: Prediction of N-glycosylation and O-glycosylation sites for 4A5S. Supplementary Table 2: Active site residues for 3WQH, 3W2T, 4A5S, 4FFW, 4PNZ and 4PV7 receptors. [file 5124165.f1.docx]

**Supplementary Files**

Supplementary Figure 1: mulPBA alignment of crystal structure similarities for 3WQH, 3W2T, 4A5S, 4FFW, 4PNZ and 4PV7 receptors. Alignment rotations at angle (A) 0˚, (B) 90˚, (C) 180˚ and (D) 270˚. 3WQH (red), 3W2T (yellow), 4A5S (green), 4FFW (blue), 4PNZ (purple) and 4PV7 (orange).


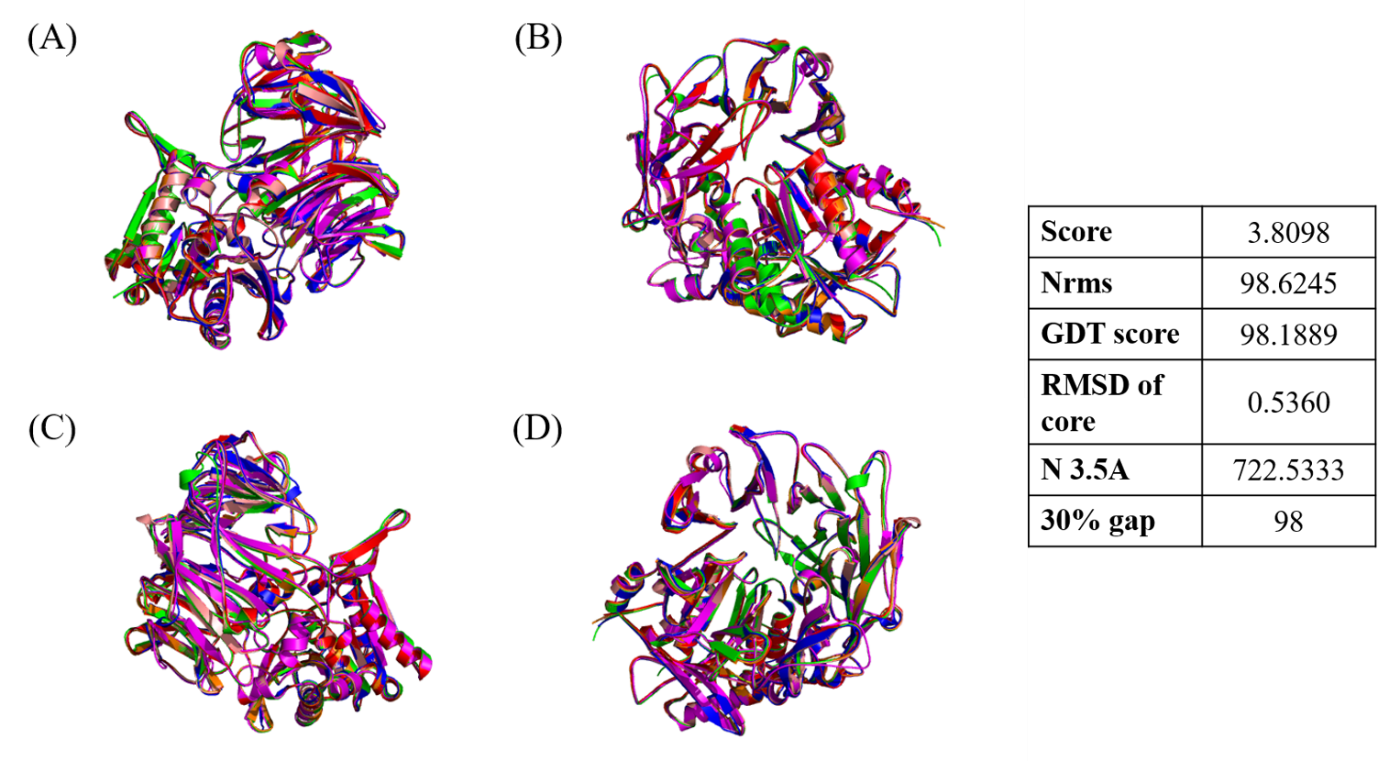


N_rms_: the percentage of alignment columns with less than 30% of elements as gaps and RMSD less than 3.0 Å; N_gdt_: the percentage of aligned positions with less than 30% gaps and maximum distance less than a given cut-off. A weighted average number of columns associated with the distance cut-offs 3.0 Å, 4.0 Å, 5.0 Å and 6.0 Å was calculated in a similar way as that of GDT score. RMSD (root mean square deviation) of core; N_3.5_: the average number of aligned residue pairs that are within a distance of 3.5 Å, counted for different combinations of pairwise comparison in the multiple alignments.

Supplementary Figure 2: Analysis results on 3WQH, 3W2T, 4A5S, 4FFW, 4PNZ and 4PV7 receptors crystal structure similarity using POSA web server. Structural alignment generated common core of 721 aa and RMSD of 0.64 Å.

(A) The superposed protein structures of PDB DPPIV receptors; 3WQH (red), 3W2T (yellow), 4A5S (green), 4FFW (blue), 4PNZ (purple) and 4PV7 (orange).


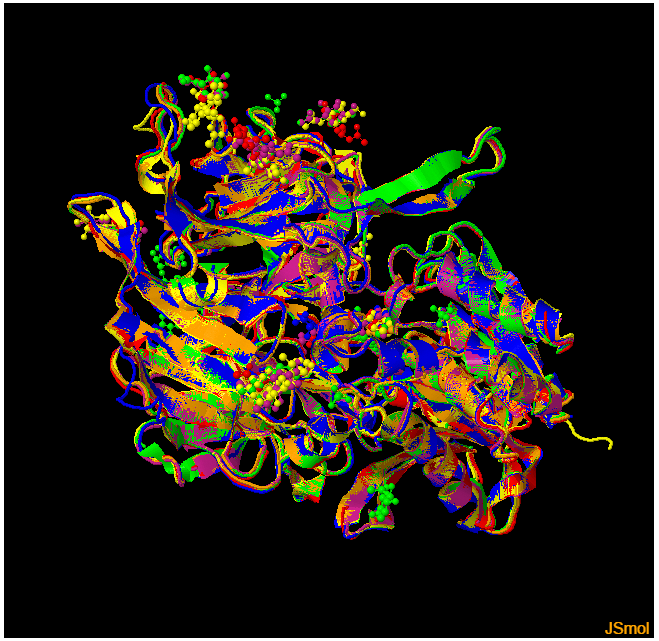


(B) Protein structures of PDB DPPIV receptors; 3WQH (red), 3W2T (yellow), 4A5S (green), 4FFW (blue), 4PNZ (purple) and 4PV7 (orange) with natural ligand positions.


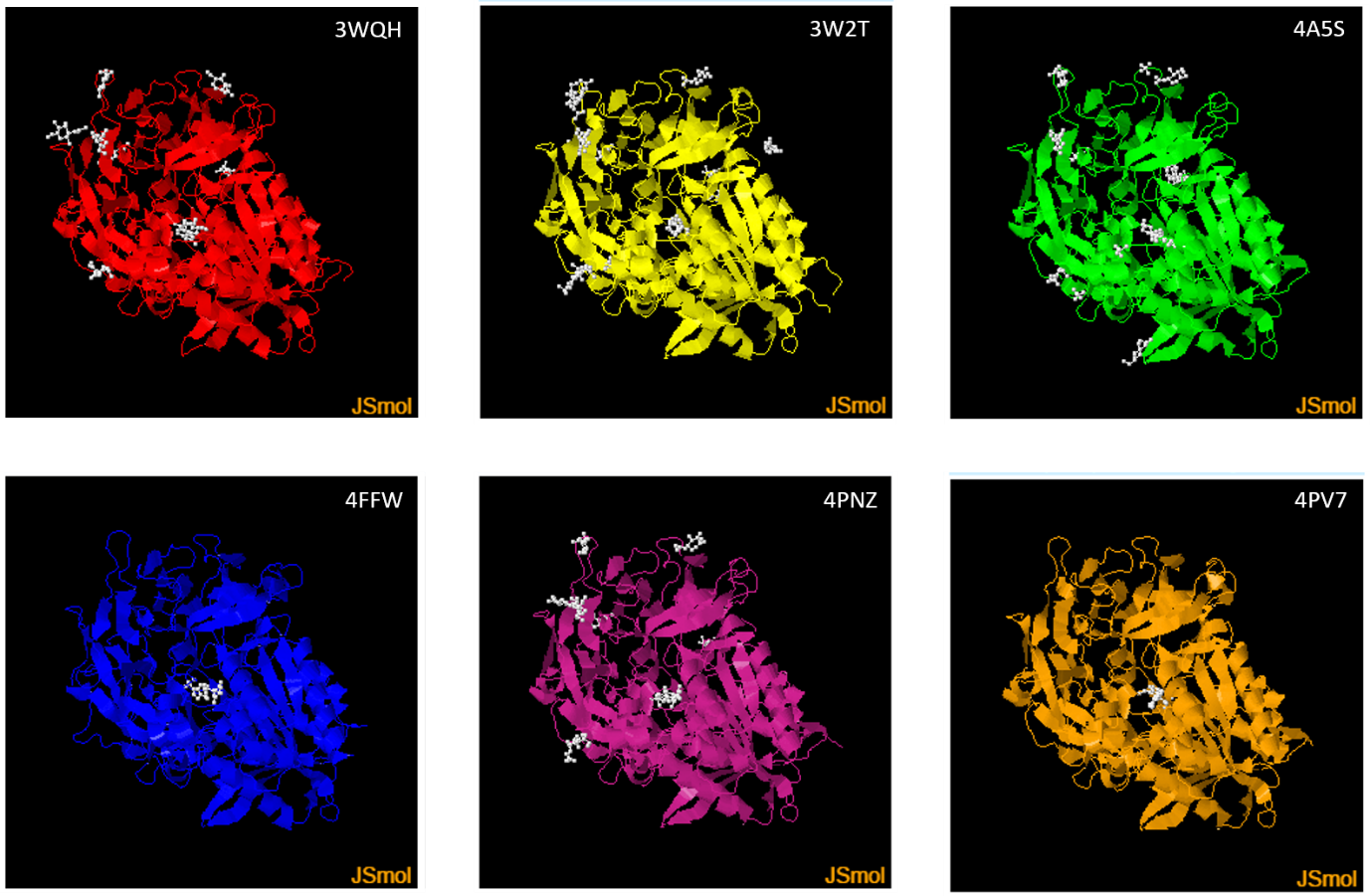


Supplementary Table 1: CASTp and SplitPocket analysis for 3WQH, 3W2T, 4A5S, 4FFW, 4PNZ and 4PV7 receptors.

| PDB ID | CASTp | | | SplitPocket | | | |
| --- | --- | --- | --- | --- | --- | --- | --- |
|  | No. Identified | Area (Å^2^) | Volume (Å^3^) | No. Identified | Length (aa) | Solvent Accesible Area (Å^2^) | Molecular Volume (Å^3^) |
| 3WQH | 197 | 5738.6 | 17325 | 47 | 143 | 4336.92 | 13724.89 |
| 3W2T | 200 | 5735.5 | 15684 | 44 | 151 | 4486.67 | 13559.75 |
| 4A5S | 184 | 6863.7 | 19238 | 41 | 181 | 5097.48 | 15039.69 |
| 4FFW | 379 | 11636.06 | 30638.25 | 25 | 145 | 4261.25 | 13043.26 |
| 4PNZ | 189 | 6318.1 | 17878 | 52 | 153 | 4543.16 | 13886.86 |
| 4PV7 | 198 | 5750.8 | 16582 | 39 | 153 | 4679.09 | 13928.94 |

Supplementary Figure 3: Prediction of N-glycosylation and O-glycosylation sites for 4A5S.

(A) Prediction of N-glycosylation sites for 4A5S.

**
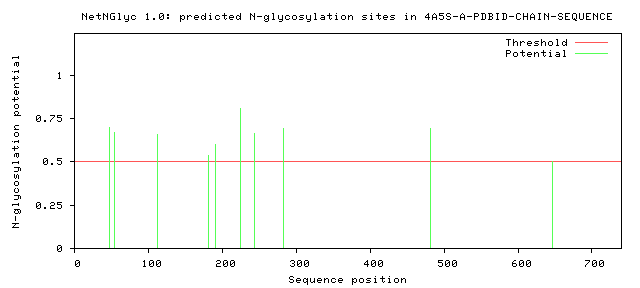
**

**(Threshold=0.5)**

**----------------------------------------------------------------------**

**SeqName Position Potential Jury N-Glyc**

**agreement result**

**----------------------------------------------------------------------**

**4A5S_A_PDBID_CHAIN_SEQUENCE 47 NSSV 0.6952 (9/9) ++**

**4A5S_A_PDBID_CHAIN_SEQUENCE 54 NSTF 0.6678 (9/9) ++**

**4A5S_A_PDBID_CHAIN_SEQUENCE 112 NNTQ 0.6592 (6/9) +**

**4A5S_A_PDBID_CHAIN_SEQUENCE 181 NGTF 0.5344 (6/9) +**

**4A5S_A_PDBID_CHAIN_SEQUENCE 191 NDTE 0.5991 (7/9) +**

**4A5S_A_PDBID_CHAIN_SEQUENCE 225 NPTV 0.8100 (9/9) +++ WARNING: PRO-X1.**

**4A5S_A_PDBID_CHAIN_SEQUENCE 243 NATS 0.6654 (8/9) +**

**4A5S_A_PDBID_CHAIN_SEQUENCE 283 NYSV 0.6931 (9/9) ++**

**4A5S_A_PDBID_CHAIN_SEQUENCE 482 NETK 0.6949 (8/9) +**

**4A5S_A_PDBID_CHAIN_SEQUENCE 647 NSTV 0.4989 (3/9) -**

**----------------------------------------------------------------------**

(B) Prediction of O-glycosylation sites for 4A5S


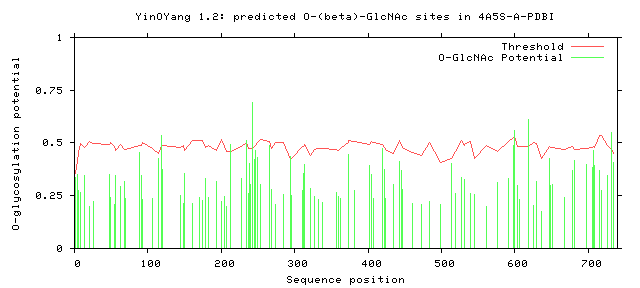


----------------------------------------------------------

SeqName Residue O-GlcNAc Potential Thresh. Thresh.

result (1) (2)

----------------------------------------------------------

4A5S_A_PDBI 118 T + 0.5346 0.4886 0.6090

4A5S_A_PDBI 213 T + 0.4928 0.4581 0.5679

4A5S_A_PDBI 235 T + 0.5138 0.5031 0.6285

4A5S_A_PDBI 242 T +++ 0.6942 0.4730 0.5880

4A5S_A_PDBI 295 S + 0.4368 0.4225 0.5199

4A5S_A_PDBI 599 S + 0.5580 0.5321 0.6676

4A5S_A_PDBI 619 S ++ 0.6129 0.4839 0.6026

4A5S_A_PDBI 732 S + 0.5497 0.4690 0.5826

----------------------------------------------------------

Supplementary Table 2: Active site residues for 3WQH, 3W2T, 4A5S, 4FFW, 4PNZ and 4PV7.

| PDB ID | Active Site Residues | No of Residues |
| --- | --- | --- |
| 3WQH | R125, E205, E206, S209, F358, R359, Y547, Y585, S630, Y631, V656, W659, Y662, D663, Y666, R669, N710, V711, H740 | 19 |
| 3W2T | R125, H126, E205, E206, S209, F357, R358, Y546, W629, S630, Y631, G632, V656, W659, Y662, D663, Y666, R669, N710, V711, H740 | 21 |
| 4A5S | R125, W201, E205, E206, S209, F357, D545, V546, Y547, G548, P550, K554, W627, GS28, W629, S630, Y631, G632, G633, V656, W659, Y662, D663, Y666, R668, N710, V711, H740, Y752 | 29 |
| 4FFW | R125, H126, W201, E205, E206, F357, R358, Y547, S630, Y631, G632, G633, V656, W659, Y662, D663, Y666, R668, N710, V711, H740 | 21 |
| 4PNZ | R125, W201, E205, E206, S209, R356, F357, R358, Y547, Y585, S630, Y631, A654, P655, V656, W659, Y662, D663, Y666, R668, N710, V711, Q715, H740 | 24 |
| 4PV7 | R125, H126, E205, E206, S209, F357, R358, Y547, P550, Y585, S630, Y631, V656, W659, Y662, D663, Y666, R668, N710, V711, H740 | 21 |
